# Correlated effects of selection for immunity in White Leghorn chicken lines on natural antibodies and specific antibody responses to KLH and *M. butyricum*

**DOI:** 10.1186/1471-2156-9-5

**Published:** 2008-01-14

**Authors:** Giulietta Minozzi, Henk K Parmentier, Sandrine Mignon-Grasteau, Mike GB Nieuwland, Bertrand Bed'hom, David Gourichon, Francis Minvielle, Marie-Helen Pinard-van der Laan

**Affiliations:** 1 INRA/AgroParisTech, UMR1236 Génétique et Diversité Animales, F-78352 Jouy en Josas, France; 2 UE de Génétique Avicole, INRA, 37380 Nouzilly, France; 3 Adaptation Physiology Group, Department of Animal Sciences, Wageningen, Institute of Animal Sciences, Wageningen University, PO Box 338, 6700 AH Wageningen, The Netherlands; 4 Station de Recherches Avicoles, Institut National de la Recherche Agronomique-Centre de Tours, 37380 Nouzilly, France

## Abstract

**Background:**

The effect of selection for three general immune response traits on primary antibody responses (Ab) to *Mycobacterium butyricum *or keyhole limpet hemocyanin (KLH) was studied in four experimental lines of White Leghorn chicken. Birds underwent 12 generations of selection for one of three different general immune criteria; high antibody response to Newcastle disease virus 3 weeks after vaccination (ND3), high cell-mediated immune response, using the wing web response to phytohemglutinin (PHA) and high phagocytic activity, measured as carbone clearance (CC). Line ND3-L was selected on ND3, line PHA-L was selected on PHA, and line CC-L on CC, but all lines were measured for all three traits. The fourth line was a contemporary random bred control maintained throughout the selection experiment. Principal component analysis was used to distinguish clusters based on the overall set of immune measures.

**Results:**

In the KLH immunised group, no differences were present between lines for natural antibodies binding to KLH and LPS, and, lines ND3-L and PHA-L had higher titers to LTA and anti-Gal titers measured before the immunisation protocol. The measure of ND3 was correlated positively with LPS titers measured post KLH immunisation and with the difference between LPS titers measured at day 0 and 7 post immunisation. In the *M. butyricum *immunised group, Line ND3-L showed significantly higher specific antibody response to *M. butyricum*, and this result agrees well with the hypothesis that the Th-1 pathway was expected to be selected for in this line.

**Conclusion:**

This study has shown that the two different antigens KLH and *M. butyricum *gave rise to different responses in the set of selected lines, and that the response was only enhanced for the antigen associated with the same response mechanism as that for the trait (ND3, PHA or CC) for which the line was selected. Interactions between innate and acquired immunity have been observed mainly for the high antibody selected trait, indicating there was a specific interaction due to the selection criterion. Furthermore, the results confirmed the independence between the three selected traits. Finally, principal component analysis contributed to visually discriminate high and low responders to the two new antigens in the four lines.

## Background

Selection for general immune response in poultry has been proposed as a sustainable alternative to selection for resistance against specific diseases, because progress with the selection for resistance approach might be hindered by interactions between host and pathogen which would lead to continuous adaptability on both sides. In addition, it would not be feasible to select for disease resistance against the tremendous number of different pathogens that an animal could face in his entire life cycle. Several general immune traits were experimentally selected for in chicken lines [1] revealing that the different immune response mechanisms may have different genetic components [2].

This study was based on three lines of White Leghorn Chickens that have been selected for 12 generations for one of three different immune response traits, high antibody response (ND3), cell mediated activity (PHA) and phagocytic activity (CC). Line ND3-L was selected on ND3, line PHA-L was selected for PHA, and line CC-L for CC, but all lines were measured for all three traits. The fourth line was a contemporary random bred Control maintained throughout the selection experiment. The results of the selection have been described by Pinard van der Laan [2]. Briefly, 200 chicks per line were hatched (800 chicks in total) in a single batch every year. Selection for each trait was done by within-family mass selection based on individual phenotype. Heritabilities estimated for the three selection criteria ND3, PHA and CC were 0.35, 0.13 and 0.15, respectively, and correlations between the traits were not significant [2]. The assessment of the disease resistance capability of the selected lines is currently under investigation and must be completed before any transposition of the results of the present work to the industry may be developed.

The question that arose from this long term selection experiment was to determine if the in-vivo selection had changed the level of other immune response traits, which is to test correlated effects, with the overall aim to investigate whether the selection was trait, antigen, mechanism or pathway specific. This might result in adding other antigens or mechanisms in the long term selection experiment. The second question was to determine whether the response for the three selection criteria had modified the levels of the humoral components of the innate immune system (natural antibodies). Finally, we were interested in estimating the associations between the immune response traits under artificial selection and the newly measured ones.

First, we investigated if the selected lines differed in their immune capabilities to mount an immune response to two other complex T-cell dependent antigens: Keyhole Limpet hemocyanin (KLH) and *Mycobacterium butyricum*, respectively. KLH is a copper-containing high molecular weight protein, found in the sea mollusc *Megathura crenulata*, which is commonly used as a soluble model protein known to induce a TH-2 like response [3]. Mycobacterium is a solubilized particulate antigen that induces a TH-1 response in rodents [4]. Significant differences were found previously between high and low chicken lines selected for SRBC (Sheep Red Blood Cells), for antibody response to *M. butyricum *[5] and for KLH [6,7], and chickens from the high antibody response line showed higher titers, irrespective of the antigen.

Secondly, the level of natural antibodies binding two different T-cell independent antigens, Lipopolysaccharide (**LPS**) and Lipoteichoic acid (**LTA**), were measured to identify if the selection had changed the levels of the humoral components of the innate immune system. Both LPS and LTA are cell wall components, and represent associated patterns of gram negative (LPS) and gram positive (LTA) bacteria, respectively.

In addition, as a further measure of the innate immunity, natural anti-Gal antibodies were measured. These antibodies represent IgM antibodies binding surface carbohydrate structures shared by a variety of pathogens [8]. Natural anti-α-Gal antibodies were measured in plasma samples by rabbit agglutinin levels, indicating antibodies reactive with Gal α 1-3Galβ-1-4GlcNAc-R, otherwise identified as the α-gal epitope. Their presence in the avian species has been previously demonstrated [9]. Chickens as well as primates lack the functional α1, 3-galactosyltransferase gene and consequently produce high levels of anti-α-Gal antibodies in response to the colonisation of the intestinal micro flora with galactosil bearing bacteria [10,11]. Previous studies on lines divergently selected for antibody titers to SRBC, demonstrated positive and moderately high correlation between RRBC (rabbit red blood cell) and SRBC titers [12]. Furthermore, a recent study reported equally high levels of anti-gal antibodies in bile of high and low diet-efficient hens [13].

In the present work, a total of 400 birds from the 12^th ^generation of selection were studied. Half of the birds were immunized with KLH and the other half with *M. butyricum *particles. Levels of Ab to all antigens were measured before and 7 or 11 days after immunization, and correlations between them were tested. Principal component analysis was performed on the measures of immune response to better visualize the association between the new measures and the three selected lines ND3-L, PHA-L and CC-L.

## Results

### Group immunized with KLH (Group A)

Mean values and results of the analyses of variance are listed in Table 1. As expected from the past selection, lines had different (P ≤ 0.001) mean values for the selected traits, but there were also significant line effects (P ≤ 0.05 to P ≤ 0.001) on four of the other measures of immunity.

**Table 1:** Immune responses (mean ± SD) and analyses of variance in the group immunized with KLH

		**Line**			
**Measure**	**Sample size**	**ND3-L**	**PHA-L**	**CC-L**	**Control**
Trait under selection					
ND3	48	5.75 ± 1.65^a^	3.00 ± 1.54^b^	1.64 ± 1.06^c^	2.56 ± 1.34^b^
PHA	48	1.13 ± 0.44^c^	1.97 ± 0.92^a^	1.34 ± 0.73^c^	1.68 ± 0.81^b^
CC	48			0.30 ± 0.07^a^	0.17 ± 0.05^b^
Before immunization with KLH (d 0)					
KLH	50	3.89 ± 1.45	4.00 ± 1.22	3.78 ± 1.23	3.69 ± 1.09
LPS	50	1.95 ± 1.73	1.85 ± 1.58	2.27 ± 1.93	1.99 ± 1.64
LTA	50	4.90 ± 1.51^a^	4.84 ± 1.43^a^	4.08 ± 1.32^b^	3.93 ± 1.23^b^
Anti-Gal	50	13.68 ± 3.82^a^	12.88 ± 3.03^a^	10.89 ± 2.92^b^	10.75 ± 2.46^b^
After immunization with KLH (d 7)					
KLH	50	8.95 ± 1.95	9.23 ± 2.24	8.04 ± 2.22	8.85 ± 2.06
LPS	49	4.49 ± 1.85^ab^	4.08 ± 1.61^bc^	3.40 ± 2.15^c^	5.02 ± 2.32^a^
LTA	48	6.12 ± 1.21^a^	5.93 ± 1.20^a^	5.17 ± 1.33^b^	5.44 ± 1.18^b^
Anti-Gal	50	20.08 ± 5.22	18.97 ± 4.15	19.64 ± 3.82	18.50 ± 3.02
		
		**Analysis of variance**			
		
		**Line**	**Sex**	**Interaction**	**R**^2^

Trait under selection					
ND3	48	***	ns	ns	0.79
PHA	48	***	ns	ns	0.72
CC	48	***	ns	ns	0.83
Before immunization with KLH (d 0)					
KLH	50	ns	ns	*	0.55
LPS	50	ns	ns	ns	0.53
LTA	50	***	ns	ns	0.64
Anti-Gal	50	**	ns	ns	0.76
After immunization with KLH (d 7)					
KLH	50	ns	ns	ns	0.71
LPS	49	*	*	ns	0.71
LTA	48	***	ns	ns	0.65
Anti-Gal	50	ns	**	ns	0.76
Responsiveness (d 7-d 0)					
KLH	50	ns	ns	ns	0.72
LPS	50	***	ns	ns	0.75
LTA	50	ns	ns	ns	0.73
Anti-Gal	50	ns	***	ns	0.75

^a-c^: Means in the same row with no common superscript differed significantly (*P *≤ 0.05) based on Duncan's multiple range test.

^1 ^Antibody titers for to KLH, LPS and LTA are the log_2 _of the reciprocal of the antibody dilution.

^2^Antibody titers to anti-Gal epitope measured in plasma samples as the agglutination titer of glutaraldehyde stabilized rabbit. Titers are expressed as the double of the column number of the last plasma dilution showing clear evidence of agglutination. Responsiveness, measured as difference between the Ab titers at day 0 and day 7 post KLH immunization

ND3-L = line selected for high antibody response to ND3 3 wk after vaccination.

PHA-L = line selected for high cell-mediated immune response, to PHA at 9 wk of age.

CC-L = line selected for high phagocytic activity, measured as carbon clearance at 12 wk of age.

Control = line selected at random

* *P *≤ 0.05, **P ≤ 0.01, ***P ≤ 0.001.

#### A. Before immunization with KLH

##### 1. The three traits under artificial selection: ND3, PHA and CC

Line ND3-L, selected for high antibody response to ND3, had the highest ND3 mean value (P ≤ 0.05). The PHA-L and Control lines had similar and intermediate mean values, and CC-L had the lowest one. The PHA response was significantly higher (P ≤ 0.05) in the PHA-L selected line, and it was similar in lines ND3-L and CC-L. CC had a higher (P ≤ 0.001) mean value in CC-L than in the Control line, and was not measured in the other lines.

##### 2. KLH, LPS, LTA and Anti-Gal titers

Natural antibodies binding to KLH and LPS did not differ significantly between the four lines, but there was a significant interaction between sex and line for natural antibodies to KLH. Antibody titers to LTA and Anti-Gal showed significant (P ≤ 0.001 and P ≤ 0.01) line differences, with higher values in lines ND3-L and PHA-L and lower ones in Lines CC-L and Control, suggesting a similar response in the ND3-L and PHA-L birds.

#### B. After immunization with KLH

##### 1. KLH, LPS, LTA and Anti-Gal titers

Specific antibody titers binding to KLH measured 7 days post immunization did not differ between the four lines. Antibody titers to LPS showed significant (P ≤ 0.05) line differences after immunization: Line CC-L had a lower response, the Control had a higher one, and the two other lines were intermediate. LTA titers differed (P ≤ 0.001) between lines, with higher means for ND3-L and PHA-L, and lower ones for the other lines. Anti-Gal titers after immunization did not show any line difference. All birds responded with increased levels of anti-Gal antibodies after immunization.

##### 2. Responsiveness of KLH, LPS, LTA and Anti-Gal titers

The only significant (P ≤ 0.001) line effect was obtained for the responsiveness of LPS, which was lower in Line CC-L. There was a significant (P ≤ 0.001) effect of the sex on the responsiveness of anti-Gal, with higher values in males from lines ND3-L, CC-L and Control (data not shown).

#### C. Principal component analysis

**Figure 1:**
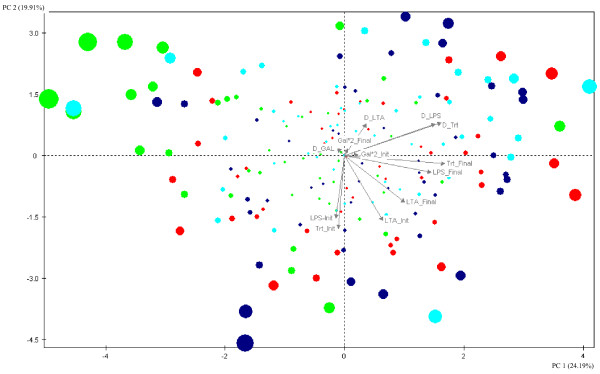
**Spread of the individuals immunized with KLH according to the principal component analysis (PCA)**. Spread of the individuals from the four lines immunized with KLH (Group A) according to the axes 1 and 2 of the principal component analysis (PCA): blue circle = line ND3-L, red = PHA-L, green = CC-L and turquoise = Control. The size of the individual circles increases with their mean contribution to the 2 axes. Trt_Init = Natural antibody titer (**Nabs**) to KLH. (Group A), Trt_Final = Antibody (**Ab**) titer after immunization with KLH, LPS-Init = Nabs to LPS at day 0, LPS_Final = Ab to LPS 7 days post immunization, LTA_Init = Nabs to LTA at day 0, LTA_Final = Ab to LTA 7 days post immunization, Gal*2_Init = Ab to anti-Gal epitope at day 0, Gal*2_Final = Ab to anti-Gal epitope 7 days post immunization, D_Trt = responsiveness of Ab response to KLH, D_LPS = responsiveness of Ab response to LPS, D_LTA = responsiveness of Ab response to LTA, D_GAL = responsiveness of Ab response to anti-Gal. PC1 = explains 24.19% of the variance. PC2 = explains 19.91% of the variance.

The results of the analysis are shown in Table 2 and Figure 1. The first five principal components explained 79.97% of the total variance. The main traits that contributed to the first axis (PC1) were KLH, LPS and LTA titers measured 7 days post immunisation, and responsiveness of KLH and LPS titers, and they were positively correlated to PC1. Axis 2 (PC2) was negatively correlated to natural antibody titers, generally representing the innate measures. The first and second principal components explained 24.19% and 19.91% of the total variance. The graph of the distribution of the 200 individuals according to PC1 and PC2 shows that individuals from the three selected lines and the control line were rather scattered and intermingled all over the plane, with no clear boundaries between lines. PC1, however, appeared to have separated CC-L somewhat towards the (upper) left part of the graph. Of all lines, the Control line was the most equally distributed over the whole area.

**Table 2:** Principal component analysis of the immune response measures of the animals immunized with KLH

	**Axis**				
	PC 1	PC 2	PC 3	PC 4	PC 5

**Variance explained (%)**	24.19	19.91	15.52	11.44	8.90

	**Eigenvector**				

Trt_Init	-0.05	-0.82	-0.03	0.20	0.05
Trt_Final	0.84	-0.09	-0.12	0.11	0.05
LPS-Init	-0.07	-0.70	-0.20	0.41	0.00
LPS_Final	0.73	-0.19	-0.13	0.28	-0.30
LTA_Init	0.32	-0.73	0.03	-0.45	-0.06
LTA_Final	0.51	-0.52	0.19	-0.14	0.48
Gal*2_Init	0.12	0.00	0.39	-0.65	0.19
Gal*2_Final	0.03	0.07	-0.79	-0.46	0.27
D_Trt	0.81	0.36	-0.08	-0.01	0.02
D_LPS	0.76	0.35	0.03	-0.04	-0.28
D_LTA	0.18	0.35	0.20	0.46	0.73
D_GAL	-0.06	0.07	-0.97	0.03	0.12

Principal component analysis of 12 measures of immune response from the 200 animals immunized with KLH (Group A): variance explained by the first 5 axes (PC1 to PC 5) and relative contributions of the 12 variables to each axis

Trt_Init = Antibody (Ab) titer before immunization with KLH, Trt_Final = Ab titer after immunization with KLH (Group A), LPS-Init = Nabs to LPS at day 0, LPS_Final = Ab to LPS 7 days post immunization, LTA_Init = Nabs to LTA at day 0, LTA_Final = Ab to LTA 7 days post immunization, Gal*2_Init = Ab to anti-Gal epitope at day 0, Gal*2_Final = Ab to anti-Gal epitope 7 days post immunization, D_Trt = responsiveness of Ab response to M.B, D_LPS = responsiveness of Ab response to LPS, D_LTA = responsiveness of Ab response to LTA, D_GAL = responsiveness of Ab response to anti-Gal.

#### D. Associations between the traits under selection and the new measures of immunity

Within-line correlation coefficients between the measures of ND3 and the other traits recorded in Group A, and the corresponding regression coefficients estimated over all lines are shown in Table 3. Antibody response to Newcastle disease virus (ND3) was correlated (P ≤ 0.05) to natural antibodies binding Anti-Gal in ND3-L. No significant correlations were found in line PHA-L. Positive (P ≤ 0.05) correlations with KLH, LTA natural antibodies and LTA titers post immunisation were found in CC-L. In the Control, positive correlations were found with the LPS titers at day 7 post-immunisation (P ≤ 0.05) and with the responsiveness of LPS titer (P ≤ 0.01). The linear regressions on LPS titers measured at day 7 and on LPS responsiveness were significant (P ≤ 0.001 and P ≤ 0.01, respectively) and positive.

**Table 3:** Associations of ND3 antibody titer with the other measures in Group A immunized with KLH

**Group A (immunized with KLH)**	**Correlation coefficient with ND3 antibody titer in Line**				
	
**Trait**	**ND3-L**	**PHA-L**	**CC-L**	**Control**	**Linear regression of ND3 antibody titer across lines**
**pha**	0.12	-0.6	-0.06	-0.26	NS
**cc**	-	-	0.15	-0.25	NS
**KLH day0**	-0.20	0.11	0.32*	-0.27	NS
**KLH day 7**	0.24	-0.10	0.22	0.12	NS
**LPS day 0**	-0.09	0.11	0.16	-0.08	NS
**LPS day 7**	0.18	0.09	0.19	0.35*	0.28***
**LTA day 0**	-0.12	-0.07	0.35*	0.01	NS
**LTA day 7**	0.05	-0.02	0.29*	-0.02	NS
**Gal day 0**	0.29*	0.00	-0.01	0.06	NS
**Gal day 7**	0.04	-0.16	-0.08	0.12	NS
**Responsiveness to KLH**	0.20	-0.15	0.05	0.24	NS
**Responsiveness to LPS**	0.25	-0.03	0.05	0.43**	0.28**
**Responsiveness to LTA11**	0.20	0.13	-0.10	-0.01	NS
**Responsiveness to Anti-Gal**	-0.19	-0.12	-0.05	0.08	NS

Negative significant correlations (P ≤ 0.05) were found in the Control line only, between the measure of PHA (T-cell response) and LTA Nabs (-0.34) and Anti-Gal antibodies (-0.36) measured after immunization. There was also a negative regression of PHA trait on Anti-Gal titer after immunisation (data not shown). Residual correlations were estimated, and the results (data not shown) confirmed the associations found by linear regression across lines.

### Group immunized with *Mycobacterium butyricum *(Group B)

Mean values and results of the analyses of variance are listed in Table 4. As in Group A, lines had different (P ≤ 0.01 or P ≤ 0.001) mean values for the traits which were under artificial selection, but a line effect (P ≤ 0.05 to P ≤ 0.001) was also present for all other traits except LPS before immunization.

**Table 4:** Immune responses (mean ± SD) and analyses of variance in the group immunized with *Mycobacterium butyricum*

		**Line**			
**Measure**	**Sample size**	**ND3-L**	**PHA-L**	**CC-L**	**Control**
Trait under selection					
ND3	48	6.10 ± 1.85^a^	2.60 ± 1.22^b^	1.75 ± 1.26^c^	2.68 ± 1.36^b^
PHA	48	1.20 ± 0.86^b^	1.87 ± 0.68^a^	1.33 ± 0.53^b^	1.43 ± 0.74^b^
CC	48			0.33 ± 0.08^a^	0.17 ± 0.04^b^
Before immunization with M.B. (d 0)					
M.B.	50	6.27 ± 1.67^a^	5.07 ± 1.47^b^	4.64 ± 1.55^b^	5.13 ± 1.13^b^
LPS	50	1.57 ± 1.54	1.45 ± 1.49	1.31 ± 1.65	2.08 ± 1.71
LTA	50	5.14 ± 1.68^a^	4.46 ± 1.43^b^	3.92 ± 1.70^b^	4.17 ± 1.75^b^
Anti-Gal	50	11.96 ± 2.98^b^	10.81 ± 2.68^b^	13.72 ± 5.81^a^	11.61 ± 3.17^b^
After immunization with M.B.(d 11)					
M.B.	50	7.73 ± 1.69^a^	7.19 ± 1.34^a^	6.29 ± 1.46^b^	6.50 ± 1.07^b^
LPS	49	2.53 ± 1.54^a^	2.26 ± 1.36^ab^	1.73 ± 1.65^b^	2.77 ± 1.82^a^
LTA	48	6.44 ± 1.54^a^	5.91 ± 1.17^ab^	5.55 ± 1.51^b^	5.51 ± 1.58^b^
Anti-Gal	50	16.48 ± 3.55^a^	14.60 ± 2.66^b^	13.72 ± 3.79^bc^	12.82 ± 3.02^c^
		
		**Analysis of variance**			
		
		**Line**	**Sex**	**Interaction**	**R**^2^

Trait under selection					
ND3	48	***	ns	ns	0.84
PHA	48	**	ns	ns	0.71
CC	48	***	ns	ns	0.78
Before immunization with M.B. (d 0)					
M.B.	50	***	***	ns	0.68
LPS	50	ns	ns	ns	0.53
LTA	50	*	ns	ns	0.69
Anti-Gal	50	**	***	***	0.82
After immunization with M.B. (d 11)					
M.B.	50	***	ns	ns	0.61
LPS	49	*	ns	ns	0.57
LTA	48	*	ns	ns	0.68
Anti-Gal	50	***	***	ns	0.77
Responsiveness (d 11-d 0)					
M.B.	50	*	***	ns	0.72
LPS	50	ns	*	ns	0.61
LTA	50	ns	**	ns	0.69
Anti-Gal	50	***	***	***	0.96

^a-c^: Means in the same row with no common superscript differed significantly (*P *≤ 0.05) based on Duncan's multiple range test.

^1 ^Antibody titers for to M.B., LPS and LTA are the log_2 _of the reciprocal of the antibody dilution.

^2^Antibody titers to anti-Gal epitope are measured in plasma samples as the agglutination titer of glutaraldehyde stabilized rabbit. Titers are expressed as the double of the column number of the last plasma dilution showing clear evidence of agglutination.

Responsiveness, measured as difference between the Ab titer at day 0 and day 11 post *M. butyricum *immunization.

ND3-L = line selected for high antibody response to ND3 3 wk after vaccination.

PHA-L = line selected for high cell-mediated immune response, to PHA at 9 wk of age.

CC-L = line selected for high phagocytic activity, measured as carbon clearance at 12 wk of age.

Control = line selected at random

*P ≤ 0.05, **P ≤ 0.01, ***P ≤ 0.001.

#### A Before immunization with *M. butyricum*

##### 1 The three traits under artificial selection: ND3, PHA and CC

Line ND3-L, selected for high antibody response to ND3, had the highest ND3 mean value (P ≤ 0.05). The PHA-L and Control lines had similar and intermediate mean values, and CC-L had the lowest one. The PHA response was significantly higher (P ≤ 0.05) in PHA-L. CC had a higher (P ≤ 0.001) mean value in CC-L than in the Control line, and was not measured in the other lines.

##### 2 *M. butyricum*, LPS, LTA and Anti-Gal titers

Natural antibody titers to *M. butyricum *showed line differences (P ≤ 0.001), and they were higher in line ND3-L than in all other lines. Titers of natural antibodies binding to LPS did not differ between lines, but titers of those binding to LTA were higher (P ≤ 0.05) in ND3-L. Measures for Anti-Gal were higher (P ≤ 0.05) in line CC-L, and an interaction between line and sex was present. Significant sex effects, but in opposite directions, were found for natural antibodies binding to *M. butyricum *and to Anti-Gal (data not shown).

#### B After immunization with *M. butyricum*

##### 1 *M. butyricum*, LPS, LTA and Anti-Gal titers

There were line differences (P ≤ 0.05 to P ≤ 0.001) for all measures of immune response after immunization. Titers of specific antibodies to *M. butyricum *were higher in lines ND3-L and PHA-L and lower in the other lines. Natural antibodies binding to LPS had higher (P ≤ 0.05) values in lines ND3-L and Control than in line CC-L. Titer was higher for LTA in ND3-L than in lines CC-L and Control. Anti-Gal measures were highest in line 1 (ND3) at day 11.

##### 2 Responsiveness of *M. butyricum*, LSP, LTA and anti-Gal titers

Responsiveness of Anti-Gal titre was higher (P ≤ 0.05) in lines ND3-L and PHA-L, and, for LPS, it was lower (P ≤ 0.05) in line CC-L than in the Control. Responsiveness was higher (P ≤ 0.05 to P ≤ 0.001) in males (data not shown) for all four measures.

#### C Principal Component analysis

**Figure 2:**
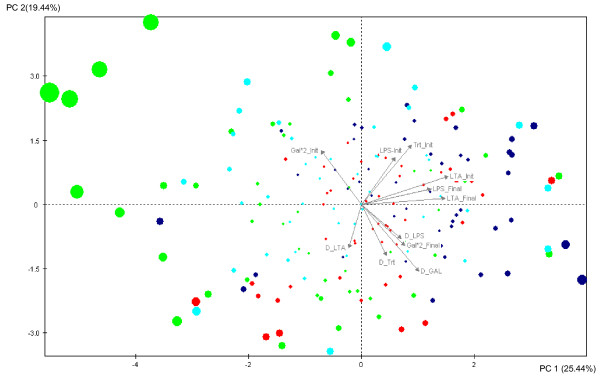
**Spread of the individuals immunized with *Mycobacterium butyricum *(Group B) according to the principal component analysis (PCA)**. Spread of the individuals from the four lines immunized with *Mycobacterium butyricum *(Group B) according to the axes 1 and 2 of the principal component analysis (PCA): blue circle = line ND3-L, red = PHA-L, green = CC-L and turquoise = Control. The size of the individual circles increases with their mean contribution to the 2 axes. Trt_Init = Natural antibody titer (**Nabs**) to M.B. (Group B), Trt_Final = Antibody (**Ab**) titer after immunization with M.B., LPS-Init = Natural antibody titer (**Nabs**) to LPS at day 0, LPS_Final = Ab to LPS 11 days post immunization, LTA_Init = Nabs to LTA at day 0, LTA_Final = Ab to LTA 11 days post immunization, Gal*2_Init = Ab to anti-Gal epitope at day 0, Gal*2_Final = Ab to anti-Gal epitope 11 days post immunization, D_Trt = responsiveness of Ab response to M.B, D_LPS = responsiveness of Ab response to LPS, D_LTA = responsiveness of Ab response to LTA, D_GAL = responsiveness of Ab response to anti-Gal. PC1 = explains 25.44% of the variance. PC2 = explains 19.44% of the variance.

The results of the analysis are shown in Table 5 and Figure 2. The first five principal components explained 76.88% of the total variance. The main contributions to the first axis (PC1) were those of the antibody titers to *M. butyricum*, the LPS titers post immunisation and both LTA titers, which were positively correlated to PC1. The second axis (PC 2) was negatively correlated to the responsiveness measures and KLH and LPS natural antibody titers. The first and second principal components explained 25.44% and 19.44% of the total variance. Figure 2 shows the distribution of the birds according to the first and second principal components. They are clearly separated along PC1 according to the line: ND3-L and CC-L are placed at the extreme positive and negative ends of the PC1 axis, and PHA-L and the Control line are centrally and similarly distributed.

**Table 5:** Principal component analysis of the immune response measures of the animals immunized with *Mycobacterium butyricum*

	**Axis**				
	PC 1	PC 2	PC 3	PC 4	PC 5

**Variance explained (%)**	25.44	19.44	11.94	10.94	9.66

	**Eigenvector**				

Trt_Init	0.45	0.62	0.12	0.22	0.42
Trt_Final	0.67	0.18	0.07	0.43	-0.23
LPS-Init	0.30	0.49	-0.55	-0.47	-0.25
LPS_Final	0.63	0.16	0.12	-0.69	-0.09
LTA_Init	0.78	0.29	-0.01	0.16	-0.06
LTA_Final	0.75	0.07	0.28	0.08	-0.15
Gal*2_Init	-0.36	0.56	0.36	0.23	-0.18
Gal*2_Final	0.39	-0.42	-0.19	0.27	0.29
D_Trt	0.22	-0.53	-0.07	0.23	-0.75
D_LPS	0.36	-0.35	0.71	-0.24	0.18
D_LTA	-0.12	-0.45	0.43	-0.22	-0.11
D_GAL	0.52	-0.69	-0.39	0.00	0.32

Principal component analysis of 12 measures of immune response from the 200 animals immunized with *M. butyricum *(Group B): variance explained by the first 5 axes (PC1 to PC 5) and relative contributions of the 12 variables to each axis.

Trt_Init = Antibody (Ab) titre before immunization with M.B. (Group B), Trt_Final = Ab titre after immunization with M.B. (Group B), LPS-Init = Nabs to LPS at day 0, LPS_Final = Ab to LPS 11 days post immunization, LTA_Init = Nabs to LTA at day 0, LTA_Final = Ab to LTA 11 days post immunization, Gal*2_Init = Ab to anti-Gal epitope at day 0, Gal*2_Final = Ab to anti-Gal epitope 11 days post immunization, D_Trt = responsiveness of Ab response to M.B, D_LPS = responsiveness of Ab response to LPS, D_LTA = responsiveness of Ab response to LTA, D_GAL = responsiveness of Ab response to anti-Gal.

#### D Associations between the traits under selection and the new measures of immunity

Within-line correlation coefficients between the measures of ND3 and the other traits recorded in Group B, and the corresponding regression coefficients estimated over all lines are in Table 6. There were positive (P ≤ 0.05) correlations with LPS day 11 titers in ND3-L and with anti-Gal responsiveness in PHA-L. Significant (P ≤ 0.05) correlations were found in CC-L for *M. butyricum *titers at day 0 and 11, LPS and LTA titers at day 11 post immunisation and LTA natural antibodies. In the Control line, the correlation (P ≤ 0.05) was positive with LTA natural antibodies and negative with LTA responsiveness. Positive coefficients (P ≤ 0.05 to P ≤ 0.001) were obtained for the regressions on LPS and LTA natural antibodies, and LPS, LTA and *M. butyricum *specific antibodies at measured at day 11 post immunization.

**Table 6:** Associations of ND3 antibody titer with the immune measures in Group B immunized with *Mycobacterium butyricum*

**Group B (immunized with *M. butyricum*)**	**Correlation Coefficient with ND3 antibody titer in Line**				
	
**Trait**	**ND3-L**	**PHA-L**	**CC-L**	**Control**	**Linear regression of ND3 antibody titer across lines**
**pha**	-0.18	-0.21	0.01	0.17	NS
**cc**	-	-	-0.08	-	NS
**MB day0**	0.021	-0.01	0.40**	0.14	NS
**MB day11**	0.17	0.01	0.46**	0.06	0.17*
**LPS day 0**	0.25	0.15	0.12	0.07	0.03*
**LPS day 11**	0.33*	0.12	0.39**	0.13	0.01***
**LTA day 0**	0.19	0.17	0.61***	0.34*	0.23***
**LTA day11**	0.21	0.21	0.45**	0.16	0.24**
**Gal day 0**	0.05	-0.22	-0.20	0.17	NS
**Gal day 11**	-0.01	0.10	0.14	0.00	NS
**Responsiveness to M.B.**	0.16	0.02	0.07	-0.09	NS
**Responsiveness to LPS**	0.04	-0.06	0.26	0.08	NS
**Responsiveness to LTA11**	-0.01	-0.08	-0.07	-0.30*	NS
**Responsiveness to Anti-Gal**	-0.04	0.29*	0.21	-0.11	NS

Negative significant correlations (P ≤ 0.001 or P ≤ 0.001) were found in the PHA-L line between the measure of PHA (T-cell response) and LTA Nabs (-0.50) and LPS (-0.46) and LTA antibodies (-0.39) measured after immunization. There was no significant regression of PHA on any of the immune traits measured (data not shown).

Residual correlations were estimated, and the results (data not shown) confirmed the associations found by linear regression across lines.

## Discussion

One of the ultimate purposes of this type of research is to be able to propose eventually a panel of a few non redundant and complementary immune variables which would best represent the general ability of the immune system to protect against infectious diseases, and could then be monitored routinely or incorporated in a selection programme. The present study was a contribution, aimed at identifying the possible correlated effects of selection for general immune traits on other, non directly selected, immune response variables. For that purpose, we tested if three lines each selected on a single immune response trait and a control line differed in immune responses to two different complex T-cell dependent antigens. The second objective of the study was to determine if the lines differed for the level of humoral components (natural antibodies) of the innate immune system. Finally we estimated correlations between the new immune measures and the selected traits.

The mean values for the traits under selection in the different lines showed similar rankings in the two groups A (KLH) and B *(M. butyricum*), indicating that the initial treatment and the sampling were done satisfactorily, and produced two initial experimental groups which were similar before they underwent the two different immunization protocols. Our results regarding Alfa-Gal Nabs, however, were not consistent at day 0 between the two groups. There is no known reason for this discrepancy which might be due to the experimental test conditions or to an unexpected sampling effect.

The humoral component of the innate immune system is made of natural antibodies the role and level of which were investigated in detail in poultry only recently [14]. Higher levels of NAbs to a variety of antigens were found in chicken lines divergently selected for high specific antibody responses to SRBC compared to the low responders [15]. This finding could suggest a genetic and/or functional relationship between the level of Nabs and the subsequent specific antibody production.

Principal component analysis was used to better understand if it was possible to distinguish clusters based on the overall set of immune measures, and then to visualize to what extent the clusters overlapped with the line of origin of the individuals.

### KLH Group

Results obtained by comparing the three selected lines and the control line, showed that after 12 generations of selection, no difference was present between lines for specific antibody titers to the new T-cell dependent antigen tested (KLH) as shown from the principal component analysis, where individuals were not forming well defined clusters in relationship to their line of origin (Figure 1). These findings are in contradiction with previous studies, where higher titers were observed in the line selected for high response to SRBC [6] compared to the low responder line. An explanation for the difference could be that the selection criterion for the ND3 response (Th-1 like induced antibody response to an attenuated vaccine) has no effect on antibody production to a strong Th-2 like inducer antigen as KLH. This is in line with the results obtained in SRBC selected lines, because the experiment was done with KLH and SRBC which both induce the Th-2 like pathway. As a consequence, it might be interesting to add the KLH response in a fourth and separated selected line in the present selection experiment to add an antigen that induces a Th-2 like response in the panel of traits.

The present results, however, are contrary to the observation made on the second generation crosses of the same lines [16] that the selected lines differed significantly from the control line for specific antibodies to KLH. Yet, KLH is a very strong Th-2 like response inducer, and conditions of immunization might have been different in the two studies. Also, favourable recombinations might have occurred in the F2 generation between genes from different selected lines. In the present study, level of Nabs binding two T-cell independent antigens (LPS and LTA) have been tested, before and after immunization with KLH. While no differences were present between lines for KLH and LPS Nabs, lines ND3-L and PHA-L had higher titers to LTA and anti-Gal titers measured before the immunization protocol. Positive and very significative correlations were found between LPS titers measured post KLH immunization and LPS responsiveness with ND3 response. In addition, we found a positive phenotypic correlation between the level of specific antibodies against KLH and NAbs to LPS in the second generation crosses of the pure lines under study [16]. Earlier, levels of antibodies to LPS were shown to be positively influenced by KLH pre-treatment [6]. These findings suggest possible co-selection of innate immunity in the line selected for specific immune response to Newcastle Disease Virus vaccine.

### Mycobacterium butyricum Group

Results obtained from principal components analysis summarise the differences observed between the selected lines for the responses to the new antigen (*M. butyricum*), the humoral innate components, and confirm the underlying correlations (Figure 2). The lines ND3-L and CC-L are located on the positive and the negative ends of the PC1 axis. The location of line ND3-L is confirmed by the ANOVA results (Table 4) since this line has higher titers for almost all immune measures which contribute most to the first axis of the PCA, except for anti-Gal Nabs and responsiveness to LTA that are both negatively correlated to this axis. The CC-L line has higher titers compared to the other lines for anti-Gal NAbs and responsiveness to LTA, and is therefore located on the left side of the figure, since the two traits are negatively correlated to the first principal component (PC1). The PHA-L line and the Control line show wider distribution of the individuals due to their intermediate values.

The line selected for ND3 showed significantly higher specific antibody response to *M. butyricum*, and this agrees well with the Th-1 pathway expected to be selected for in line ND3-L and the Th-1 like response induced by *M. butyricum *[4]. Previous studies on commercial chickens differing in residual feed intake (RFI) demonstrated no difference between the efficient and inefficient animals in specific antibody production to *M. Butyricum*, suggesting that this response does not influence feed efficiency [17] and confirming that antibody production is a not an energy demanding process [18,19]. This experiment was focused on layers, however, and it would be interesting to investigate the effect of selection for immune traits on other production traits and for other types of poultry. The positive correlations between ND3 and natural antibodies to LPS and LTA in the *M. butyricum *treatment group suggest a possible co-selection of the innate immune responses. They confirm also the results obtained in the second generation crosses of the same pure lines [16]. In lines divergently selected for SRBC, correlations between primary antibody response to SRBC and natural antibodies to LPS were low but significant [6]. Recently, levels of natural antibodies and specific antibodies to Newcastle disease virus (NDV) have been measured in 12 purebred lines of laying hens in relation to their survival rate. Results showed no correlation between innate and acquired immune response although the lines that had a higher response of antibodies to NDV showed also higher levels of natural antibodies [20].

Comparing responses in KLH- versus *M. butyricum *challenged birds, it is noteworthy that line differences in the KLH treated birds were found in the day 0 samples. This suggests that the selection for the three immune traits changed the immune status in the 'non-challenged' birds. All lines responded equally to KLH on the level of Nabs and specific antibodies, which might be due to the strong Th2 nature of the antigen (KLH) that overrules the selection criteria.

Otherwise, the current data suggest that, regardless of selection, the lines can mount compensatory responses as illustrated by the anti-Gal responses. It is clear also that the *M. butyricum *challenge revealed more line differences than KLH for the three measures of immune response selected in the pure lines and for correlated traits, possibly because of the nature of *M. butyricum *itself and of the strong Th-1 like response it induces.

## Conclusion

This study has shown that the immune response induced by the *M. Butyricum *and KLH antigens, associated respectively to the Th-1 and Th-2 pathways, was not similar in the three selected chicken lines. In one case, the differences between lines were marked (*M. butyricum*) and in the other one (KLH) they were absent, indicating that an associated effect of selection in the pure lines had been to enhance the immune response based on the same mechanism than that of the selected trait, as was the case for *M. butyricum *and ND3 (Th-1 pathway), but not for KLH. As a consequence, Ab titers to KLH could be used as a measure of immune response associated to the Th-2 pathway in a new selected line developed to take advantage of this pathway. In the framework of the usual poultry (or swine) systems of production, a four-way commercial cross between these four lines selected for complementary immune response might then benefit from expanded general immunity. Interactions between innate and acquired immunity have been observed mainly for the ND3 trait, indicating there is a specific interaction due to the selection criterion and confirming the independence between the three selected traits. In addition, through PCA analysis it was possible to discriminate visually high and low responders to all the antigens tested, and this approach might be explored further for ranking potential breeders on immune response, in order to select them on overall immunity rather than on each specific trait one at a time.

More attention should be given to the correlation between immune response, production traits and disease resistance. No differential mortality or reproduction performances were observed in the three lines under selection, but selection seemed to have some effect on body weight (data not shown). Research on the effects of selection for immune response on disease resistance is currently under way in the three selected pure lines.

## Methods

### Animals and selection procedure

The selected lines were developed by 12 generations of selection from a cross between an experimental White Leghorn line segregating for the sex-linked dwarf (*dw*) gene and a commercial Babcock^® ^White Leghorn line. Three immunity-related traits were measured on all birds, at each generation, but each line was under selection for one of them only: high antibody response to Newcastle disease virus (HB1 vaccine) 3 weeks after vaccination (ND3) in L1 (ND3-L), high cell-mediated immune response, using the wing web response to PHA at 9 weeks of age in L2 (PHA-L), high phagocytic activity, measured as clearance of carbon at 12 weeks of age in L3 (CC-L), and random selection in L4 (Control). Every year, all birds were hatched in a single batch, and 15 males and 30 females were chosen as breeders out of 100 candidates per sex and line, by within-family mass selection based on individual phenotypic performance. Mating was at random, but full and half sib matings were prohibited.

### Housing

After hatching, chicks were housed and lines intermingled, in a three-tier battery of group cages, in which they remained until the end of the experiment. Each cage housed about 13 animals. Both sexes were kept in separate cages but housed in the same room. Artificial light was 16 h/d. The birds were fed a layer diet (2,685 kcal ME/kg and 175 g/kg crude protein) *ad libitum*, with free access to water. Birds were vaccinated against Marek's disease at hatch by intramuscular injection; spray vaccinated for infectious bronchitis at d 1 and 63; against Gumboro disease via drinking water at d 19, 37 and 84; against Newcastle disease by intraocular way (eye drop) at d 26 and in drinking water at d 62; against Avian encephalomyelitis via drinking water at d 98, and spray-vaccinated foravian infectious Rhinotracheitis or Swollen Head Syndrome at d 77. All birds were weighed at 8 wk of age.

### Experimental design

The treatment consisted in an immunization protocol with KLH Keyhole Limpet Hemocyanin (group A) or with *M. butyricum *(group B) which started when the animals were 14 weeks of age. Each treatment group was composed of 200 birds, 50 per line (3 selected lines and 1 control line) belonging to 12^th ^generation of selection. The two groups A and B were designed to be as similar genetically as possible, by choosing for each line and in each full sib family two animals of the same sex to be assigned to each group. Blood samples were taken on day 0 (at 14 wk of age, before primary immunization) and 7 days post immunization with KLH (group A) and 11 days post immunization with *M. butyricum *(group B). Immunization doses and days of sampling were based on previous studies on selected chicken lines [7]. Responsiveness was measured for the four non-selected immune traits as the difference between antibody titer at day 7 (after immunization with KLH) or day 11 (after immunization with *M. butyricum*) and antibody titer at day 0.

### Humoral Immune response essay

Total immunoglobulin titers to KLH (Sigma-Aldrich GmbH, Schnelldorf, Germany), and levels of NAB binding to Salmonella Enteriditis Lipopolysaccharide LPS(Sigma Chemical Co., St Louis, MO) and to Lipoteichoic acid LTA and KLH in plasma of 200 birds previously immunized at 14 wk of age with 1 mg/ml of KLH were measured by an indirect two step ELISA procedure at day 0 and d 7 post immunization. Total antibody titers binding to *M. butyricum *and levels of NAB binding Salmonella Enteriditis Lipopolysaccharide LPS(Sigma Chemical Co., St Louis, MO) and Lipoteichoic acid (LTA) were measured in plasma samples of 200 birds previously immunized with 1 mg/ml of *M. butyricum *protein (Difco, Laboratories, Detroit, USA) at day 0 and day 11 days post immunization. Briefly, 96 well plates were coated either with 1 μg/mL of KLH, 4 μg/mL of *M. butyricum*, 10 μg/mL of LTA or 4 μg/mL of LPS.

After washing with tap water and 0.05% Tween, plates were incubated with serial dilutions of plasma. Binding of total antibodies to KLH, *M. butyricum *and of natural antibodies to LPS, LTA, KLH and *M. butyricum *was detected using 1:20,000 diluted rabbit anti chicken (IgG_H+L_)(Nordic, Tilburg, The Netherlands) coupled to peroxidase (PO). Tetramethylbenzidine and 0.05% of H_2_0_2 _were added and incubated for 10 min at room temperature. The reaction was stopped with 1.25 *M *of H_2_SO_4 _and absorbencies were measured with a Multiscan (Labsystems, Helsinki, Finland) at a wavelength of 450 nm. Titers were expressed as the log_2 _values of the highest dilution giving a positive reaction. Titers were derived from the absorbance values of a positive control serum present on every microtiter plate.

### Anti-Gal Antibody Determination

Anti-Gal antibody titer was determined by an agglutination test to rabbit red blood cells (RRBC) because these cells express high levels of the α-Gal epitope [12]. The test was performed in 96-well U-bottom plates. Plasma samples were added to the first and second wells (25 μl), and 25 μl of PBS were then added to columns 2 to 11. Next, serial dilutions (1:2) were made from column 2 to 11. Column 12 contained only 25 μl of PBS, as a negative control. Then, 25 μl of a 1% solution of rabbit red blood cells were added to each well. Plates were vortexed gently for a few seconds and incubated overnight at room temperature. Titers were recorded as the column number of the last plasma dilution showing clear evidence of agglutination.

### Statistical Analysis

Plasma Ab titers to KLH, ND3, *M. butyricum*, skin swelling to PHA, and levels of NAB binding LPS, LTA and Anti-Gal were analyzed by a mixed 3-way ANOVA with the sex and the line as fixed effects, the sire as a random effect nested within the line, and with all possible interactions. All analyses were done with the GLM procedure of SAS [21]. The sire mean square error was used to test the overall significance of the effect of the line. The mean square error of the interaction between sex and sire within line was used to test the significance of the effect of sex and of the interaction between the sex and the line. When a main effect was found to be significant, the corresponding line means were compared by using Duncan's multiple range test. Phenotypic correlations were computed within line, and linear regression coefficients have been estimated across all lines. In addition, residuals obtained from adjusting a linear model including the effects of line, sex and interaction were obtained for each measure of immunity, and their correlations (residual correlations) were estimated.

### Principal Component Analysis

Principal component analysis (PCA) was applied to the entire dataset and was performed with the SPAD 6.0 software [22]. The normality of each measure was checked using UNIVARIATE procedure of SAS [21] because PCA is only used for measures normally distributed, and for individuals measured for several traits. The objective of this analysis is to find a small number of factors (the "principal components") which are linear combinations of the original variables, and which best explain the total variation between animals. Each variable is associated to a principal component by an eigenvector and an eigenvalue, which indicate respectively the contribution and the correlation of the trait to the specific principal component axis.

## Authors' contributions

GM designed the study, wrote the paper, collected samples and performed the statistical analysis. HP participated in the design of the experiment, the interpretation of the results and commented on an earlier draft of the paper. SMG performed the principal component analysis. MN and GM carried out the immunoassays. BB assisted in the technical aspects of the immunization protocols and commented on earlier drafts of the paper. DG supervised, organised and carried out the sample collection. FM assisted and contributed in the statistical analysis, the writing of the paper and suggested the use of the principal component analysis. MHP participated in the coordination and design of the study and commented on earlier drafts of the paper.
